# The Mechanism of Cardiac Sympathetic Activity Assessment Methods: Current Knowledge

**DOI:** 10.3389/fcvm.2022.931219

**Published:** 2022-06-23

**Authors:** Jiakun Li, Lihui Zheng

**Affiliations:** National Center for Cardiovascular Diseases, Fuwai Hospital, Chinese Academy of Medical Sciences and Peking Union Medical College, Beijing, China

**Keywords:** cardiac sympathetic activity assessment, sympathetic innervation, sympathetic regulation, mechanism, stellate ganglion

## Abstract

This review has summarized the methods currently available for cardiac sympathetic assessment in clinical or under research, with emphasis on the principles behind these methodologies. Heart rate variability (HRV) and other methods based on heart rate pattern analysis can reflect the dominance of sympathetic nerve to sinoatrial node function and indirectly show the average activity level of cardiac sympathetic nerve in a period of time. Sympathetic neurotransmitters play a key role of signal transduction after sympathetic nerve discharges. Plasma or local sympathetic neurotransmitter detection can mediately display sympathetic nerve activity. Given cardiac sympathetic nerve innervation, i.e., the distribution of stellate ganglion and its nerve fibers, stellate ganglion activity can be recorded either directly or subcutaneously, or through the surface of the skin using a neurophysiological approach. Stellate ganglion nerve activity (SGNA), subcutaneous nerve activity (SCNA), and skin sympathetic nerve activity (SKNA) can reflect immediate stellate ganglion discharge activity, i.e., cardiac sympathetic nerve activity. These cardiac sympathetic activity assessment methods are all based on the anatomy and physiology of the heart, especially the sympathetic innervation and the sympathetic regulation of the heart. Technological advances, discipline overlapping, and more understanding of the sympathetic innervation and sympathetic regulation of the heart will promote the development of cardiac sympathetic activity assessment methods.

## Introduction

The cardiac autonomic nervous system (ANS) is one of the most significant structures of the neurohumoral system that regulates cardiac function. ANS can be divided into the sympathetic nerves and the parasympathetic nerves depending on the composition of the nerves (1, 2). A vast number of research studies have proved that sympathetic nerve dysfunction engages in the pathophysiological process of coronary heart disease, hypertension, heart failure, arrhythmia, etc. (3, 4). Consequently, the evaluation of autonomic nervous activity is in favor to understand the regulation of cardiovascular activity and the pathogenesis of cardiovascular disease (CVD). Meanwhile, the clinicians can assess treatment response, progression, and risk of recurrence in patients with CVD depending on the evaluation of autonomic nervous activity (5–7). In this review, we discussed the methods of cardiac sympathetic activity assessment applied in the clinical or under research with emphasis on the mechanism beneath these approaches, aiming at a thorough understanding of current sympathetic activity assessment and further exploration.

## Neuroanatomy and Neurophysiology of the Heart

### Sympathetic Innervation of the Heart

The ANS is divided into the sympathetic and parasympathetic subsystems, controlled by regulatory centers in the midbrain, hypothalamus, pons, and medulla. The cardiac sympathetic nerve center is located on the medial lateral column of the first to fifth thoracic segment of the spinal cord. The cardiac sympathetic innervation consists of extrinsic and intrinsic components according to anatomical position (8, 9). The extrinsic sympathetic nerve comes from the superior cervical ganglia and the cervicothoracic (stellate) ganglia, which, respectively, connect with the cervical nerves C1–C3 and with the cervical nerves C7–C8 to the thoracic nerves T1–T2 (10). In addition, the thoracic ganglia (as low as at least the fourth thoracic ganglion) also contribute to the sympathetic innervation of the heart (11). These ganglia hold the cell bodies of most postganglionic sympathetic neurons whose axons form the superior, middle, and inferior cardiac nerves and terminate on the surface of the heart (3). These descending sympathetic neurons’ postganglionic fibers reach the surface of the heart, communicate with each other, and form nerve fibers network and ganglion plexuses, which constitute the intrinsic sympathetic nerve of the heart (12). In addition, sympathetic postganglionic fibers, which originate from the superior cervical and stellate ganglion, are widely distributed in the skin of the upper limb and chest (13).

### Sympathetic Regulation of the Heart

Cardiac sympathetic nerves play a vital role in regulating sinoatrial node, atrioventricular node, and activity of the segmental myocardium, which depend on neurotransmitters [norepinephrine (NE), dopamine, etc.] released by synapses at sympathetic nerve terminals and NE receptors on the cell membrane (14–16).

Norepinephrine is mainly synthesized by tyrosine hydroxylase at the sympathetic nerve terminals and stored in the vesicles (15). Once the sympathetic nerves are triggered, NE will be emitted and bound to the receptors on the cell membrane of sinoatrial node, atrioventricular node, and myocardium, which, respectively, increases heart rate, enhances atrioventricular node conduction, accelerates the repolarization of myocytes, and strengthen contractile ability of myocardium (14, 16, 17). Jung et al. uncovered that an increase in stellate ganglia sympathetic activity is followed by an increase in heart rate, and the circadian rhythms of heart rate are highly consistent with circadian rhythms of cardiac sympathetic nerve activity (18). After signal transduction, much of the NE will be reabsorbed by sympathetic nerve terminals, while only a little NE will get into circulation and be inactivated in the liver and kidney (19).

## Cardiac Sympathetic Activity Assessment

Nowadays, the methods of cardiac sympathetic activity assessment applied in the clinical practice or under research are based on the anatomy and physiology of the heart, especially the sympathetic innervation and the sympathetic regulation of the heart.

### Heart Rate Variability Analysis

Heart rate variability originates from the study on the heart rate patterns and cardiac rhythms, which could date back to 1965. In the 30 years since more and more clinicians had recognized the physiological and pathological significance of HRV. Until 1996, the European Society of Cardiology and the North American Society of Pacing and Electrophysiology published the standard of measurement, physiological interpretation, and clinical use of HRV (20, 21).

Heart rate variability relies on the analysis of every heartbeat, which is directly controlled by sympathetic and parasympathetic activities. Consequently, analyzing the patterns of heart rate, more precisely, analyzing beat-to-beat changes in the R-wave to R-wave intervals can indirectly reflect and evaluate the overall balance state of the cardiac autonomic nerve (21, 22). HRV analysis includes time-domain analysis, frequency-domain analysis, and non-linear analysis (Table 1). Time-domain analysis quantifies the amount of HRV observed during monitoring periods that may range from 5 min to 24 h. Frequency-domain values calculate the absolute or relative amount of signal energy within component bands. Non-linear measurements quantify the unpredictability and complexity of a series of interbeat intervals (23, 24).

**TABLE 1:** Overview of heart rate variability (HRV) analysis for sympathetic nerve activities.

Variable	Units	Description	Correlation with autonomic activities
**Time domain analysis**			
SDANN	ms	Standard deviation of the averages of NN intervals in all 5 min segments of the entire recording	negative correlation with sympathetic nerve
SDNNI	ms	Mean of the standard deviations of all NN intervals for all 5 min segments of the entire recording	negative correlation with sympathetic nerve
SDNN	ms	Standard deviation of all NN intervals	the balance between the sympathetic nerve and parasympathetic nerve
HRV triangular index		Total number of all NN intervals divided by the height of the histogram of all NN intervals measured on a discrete scale with bins of 7.8125 ms (1/128 s)	negative correlation with sympathetic nerve
**Frequency domain analysis**			
Very low frequency	ms^2^	Power in the very low frequency range	efferent sympathetic nerve activities
Low frequency	ms^2^	Power in the low frequency range	sympathetic nerve (< 0.1 Hz) and parasympathetic nerve activities
LF norm	n.u.	LF power in normalized units	
High frequency	ms^2^	Power in the high frequency range	parasympathetic nerve activities
HF norm	n.u.	HF power in normalized units	
LF/HF		Ratio LF (ms^2^)/HF (ms^2^)	the ratio between sympathetic nerve and parasympathetic nerve activities
**Non-linear measurements**			
S	ms	area of the ellipse	total HRV
SD1	ms	Poincaré plot standard deviation perpendicular the line of identity	SD1 predicts diastolic BP, HR Max – HR Min, RMSSD, pNN50, SDNN, and power in the LF and HF bands, and total power during 5 min recordings
SD2	ms	Poincaré plot standard deviation along the line of identity	SD2 measures short- and long-term HRV in ms and correlates with LF power
Approximate entropy		brief time series in which some noise may be present	measurement of the regularity and complexity of a time series
DFA		the correlations between successive RR intervals over different time scales	description of short- or long-term fluctuations

Time domain analysis is used to measure and analyze the variability of the R-R interval of sinus rhythm by statistical and geometric methods (Table 1). Among the commonly used indexes, mean standard deviation (SDANN, estimate of long-term components of HRV), mean standard deviation index (SDNNI), and HRV triangular index (estimate of overall HRV) can reflect the sympathetic nerve tension. The smaller the value is, the greater the sympathetic nerve tension is. The overall SD (SDNN, estimate of overall HRV) reflects the balance between the sympathetic nerve and parasympathetic nerve (20, 22). In clinical practice, low HRV, which implicates increased sympathetic activity, is associated with a poor prognosis of CVDs, such as myocardial infarction (MI) and heart failure. In a randomized, double-blind control study of 3,717 patients with postmyocardial infarction and depressed left ventricular function, Camm found that low HRV (HRV triangular index ≤ 20 baseline width unit) independently identified a subpopulation at high risk of mortality (25, 26).

Frequency-domain analysis is to analyze the spectrum curve formed by the R-R interval time series signal of sinus rhythm (Table 1). The spectrum curve obtained by power spectral density (PSD) analysis provides the basic information on how power (i.e., variance) distributes as a function of frequency (20, 21). Usually, spectral analysis, calculated by taking a 5-min electrocardiograph (ECG) recording, includes three main spectral components: very low frequency (VLF, ≤ 0.04 Hz), low frequency (LF, 0.04–0.15 Hz), and high frequency (HF, 0.15–0.4 Hz) components. LF is mainly related to sympathetic activity and the low-frequency and high-frequency power ratio (LF/HF) is correlated to the ratio between sympathetic nerve and parasympathetic nerve activities. The central frequencies of LF and HF are not fixed but vary with the modulation of ANS to the cardiac rhythm. Therefore, the normalized LF [LF power in normalized units, LF/(Total Power − VLF) × 100] and HF [HF power in normalized units, HF/(Total Power − VLF) × 100] could be more valuable (20–22). Frequency-domain measures of heart period variability can also be evaluation indicators of CVDs, such as MI, hypertension, and heart failure (27, 28).

Time- and frequency-domain analyses of HRV are fairly simple and stable methods for sympathetic nerve activity assessment. However, these methods could not extract key information from complex interactions of hemodynamic, electrophysiological, and humoral variables, as well as by autonomic and central nervous regulations, called non-linear phenomena (20, 21). Hence, analysis of HRV based on the methods of non-linear dynamics (i.e., non-linear measurements) might elicit valuable information for the physiological interpretation of HRV (Table 1). Non-linear measurements are achieved by plotting every R-R interval against the prior interval, creating a scatter plot called Poincaré plot (return map). S (area of the ellipse which represents total HRV), SD1 (Poincaré plot SD perpendicular to the line of identity), SD2 (Poincaré plot SD along the line of identity) are commonly used indices. Detrended fluctuation analysis (DFA), extracting the correlations between successive R-R intervals over different time scales, could analyze a time series that spans hours of data. Approximate or sample entropy could give a judgment to the predictability of fluctuations in successive R-R intervals (20, 29). Gronwald found that DFA performs well in the analysis of complex autonomic activity at rest or during intense exercise. Moreover, Boos et al. found that non-linear HRV is more sensitive to the effects of high altitude than time- and frequency-domain indices. These proofs indicate that non-liner has more potential application prospect in complex or untraditional situations (30–32).

The application of HRV analysis is not only limited to CVDs, but also can be applied to obesity, tumor, and other diseases with autonomic nervous disorders (33, 34). While HRV is influenced by a number of physiological and pathological factors. Awareness of these mediators or confounders is of great importance in the analysis and assessment of HRV both in scientific studies and in clinical practice (Table 2). In the clinical use of HRV, age, gender, and ethnic origin should take into consideration firstly. In addition, diseases (sepsis, lung diseases, metabolic diseases, and psychiatric diseases) and internal and external factors (smoking or increased body weight, sporting activity, alcohol abuse, noise, medications, night shift work, or harmful substances) may also exert influence on HRV (6, 35).

**TABLE 2:** Comparison of cardiac sympathetic nerve activities assessment methods.

Methods	Theoretical cornerstone	Measurement	Indices	Clinical interpretation	Periods of time	Advantages	Disadvantages
HRV	sympathetic nerve activities regulation of Sinus node	ECG recording	See Table 1	sympathetic activities, balance between sympathetic and parasympathetic nerve activities	several minutes to 24 h	simple, non-invasive, stable	influenced by many factors
plasma catecholamine detection	release of neurotransmitters after sympathetic excitation	peripheral or coronary sinus blood	plasma catecholamine concentration	global sympathetic activities	a few minutes	simple, easy to interpret	inaccuracy in cardiac sympathetic activities assessment
cardiac neurotransmission imaging	release of neurotransmitters after sympathetic excitation	radiolabeled neurotransmitter analogues, SPECT and PET	Heart-to-mediastinum ratio (HMR) and mIBG wash-out (WO) rate	uptake and retention of norepinephrine	a few minutes	Location information of abnormal cardiac sympathetic activities	invasive, expensive
SKNA	detection of stellate ganglion discharge from the skin surface	ADInstruments PowerLab or ME6000 portable biomonitor with increased bandwidth and sampling rate	average SKNA and SKNA burst	average sympathetic nerves activity, number of sympathetic nerves burst discharges after excitation during selected period	instant, several minutes to 24 h	simple, non-invasive, continuous evaluation	lack of a complete evaluation system and reliable reference value

Besides HRV, blood pressure variability, resting heart rate, and so on are also closely related to the state of ANS, which, therefore, are available to evaluate cardiac sympathetic activity (36, 37).

### Sympathetic Neurotransmitters Detection

Sympathetic neurotransmitters, released by synapses at sympathetic nerve terminals, are the transmitter between the sympathetic and the heart, which could be the target of cardiac sympathetic activity detection (38, 39).

Most of the sympathetic neurotransmitters, such as NE, dopamine, and epinephrine, will be reabsorbed by sympathetic nerve terminals after signal transduction, but there will also be a little NE getting into circulation and can be detected by a peripheral blood test (40, 41). Plasma catecholamine levels are normally positively correlated to sympathetic activities (Table 2). Moreover, William et al. (41) found that exercise could cause an increase in plasma catecholamine level, with a precipitous drop in the levels at 5 min of recovery. In addition, the more intense the exercise, the higher the plasma catecholamine level. However, it is worth noting that plasma catecholamine level reflects the sympathetic activities of the whole body, not specifically referring to cardiac sympathetic activities (42). Measurement of the coronary sinus and arterial blood catecholamine concentrations can be a possible solution to estimate transcardiac NE despite its possible surgical risks. Kaye et al. found that arterial and transcardiac NE are significantly higher in heart failure with preserved ejection fraction patients than controls (43). However, the risks of interventional surgery make it difficult to implement for general patients.

The measurement of local catecholamine levels seems quite challenging, while radio imaging combined with cardiovascular physiology makes it possible to precisely detect cardiac local sympathetic neurotransmitters at a micromolar level (44). Applying iodine-123 meta-iodobenzylguanidine (^123^I-mIBG) or other radiolabeled neurotransmitter analogs, cardiac neurotransmission imaging with single photon emission computed tomography (SPECT) and positron emission tomography (PET) allows *in vivo* assessment of presynaptic reuptake and neurotransmitter storage and of regional distribution and activity of postsynaptic receptors (Table 2) (5, 44). Heart-to-mediastinum ratio (HMR) and mIBG wash-out (WO) rate are the most commonly used scientific parameters. HMR is the indicator of mIBG uptake, and retention of NE by sympathetic neurons can be semiquantified by WO rate, specifically, comparing early and delayed activities (45). Yasushi et al. found that lower HMR was the independent predictor of the transit from idiopathic paroxysmal atrial fibrillation (AF) to permanent AF, manifesting the fact that cardiac sympathetic nerve activity abnormal plays a key role in the development of atrial fibrillation (46).

Poor imaging quality and difficulty in distinguishing different cardiac structures were the main problems that limited the application of this technique for a long time in the past. However, the recent development of solid-state gamma camera technology with significantly improved sensitivity, spatial resolution, and energy resolution has enabled high-quality SPECT imaging with a spatial resolution of ≤ 5 mm (44, 47). In addition, the injection of radiolabeled neurotransmitter analogs and their possible radiation damage may cause concern among patients and block their clinical use (44).

### Sympathetic Nerve Activity Recording

A stellate ganglion can directly regulate the activity of the cardiac sympathetic nerve and then regulate cardiac activity. Enhanced discharge activity of stellate ganglion can accelerate heart rate and raise blood pressure (48). Sympathetic nerve activity record of the heart is on the basis of sympathetic nerve innervation using neuroelectrophysiological methods, which went through three stages of exploration——stellate ganglion nerve activity (SGNA), subcutaneous nerve activity (SCNA), and skin sympathetic nerve activity (SKNA) (18, 49–51).

#### Stellate Ganglion Nerve Activity

Lavian et al. (52) directly placed recording electrodes on the surface of canine stellate ganglion nerve fibers to record the discharge of stellate ganglion after thoracotomy and can record the nerve activity in living dogs within 1 min. Jung et al. (18) further improved the method, so that SGNA recording can complete recording lasting more than 40 days for 24 h. It is found that after SGNA recording showed the discharge signal of the stellate ganglion, the heart rate and blood pressure of dogs were increased secondary. At the same time, the discharge of stellate ganglion shows circadian rhythm, which is consistent with the circadian rhythm of heart rate (18). Subsequently, Tan et al. (53) found that the occurrence of arrhythmia diseases, such as atrial tachycardia, ventricular tachycardia, and atrial fibrillation, is related to the abnormal discharge of stellate ganglion through the SGNA recording of dog model, and the discharge patterns of stellate ganglion are also different for different arrhythmia diseases. In the SGNA recording of complex CVDs, such as MI, heart failure, and sudden death, Zhou et al. (54) found that the increase of discharge activity of stellate ganglion is an important reason for the progression of ventricular arrhythmia and other CVDs (55, 56). These findings provide scientific evidence for understanding the changes in cardiac sympathetic nerve activity in the occurrence and development of CVDs and finding appropriate treatment methods (57, 58).

Stellate ganglion nerve activity recording can be recorded continuously for more than 40 days for 24 h in living animals. It can record the immediate discharge activity of stellate ganglion without affecting the survival and daily activities of animals. It is an important tool to study the cardiac sympathetic nerve activity (57, 58). However, SGNA recording is carried out by thoracotomy, which has great trauma and is difficult to be routinely applied in the clinic. In order to reduce the trauma caused by recording, a new recording method, SCNA recording, has been published (49).

#### Subcutaneous Nerve Activity

In addition to innervating the heart, some postganglionic fibers of the stellate ganglion are widely distributed in the skin and subcutaneous tissue of the neck and chest, and there is extensive cross-linking in the whole neural network (49, 59). Robinson et al. (49) speculated that when the stellate ganglion discharges, the stellate nerve postganglionic fiber terminals of the skin and subcutaneous tissue of the neck and chest appear synchronous discharge.

Robinson et al. (49) implanted the recording electrode into the subcutaneous tissue of the dog’s chest to record the SCNA and performed SGNA recording by thoracotomy. The results showed that before the dog’s heart rate accelerated, SGNA and SCNA recording showed synchronized neural discharge activities, and the 24 h recording results showed that SGNA recording and SCNA recording had consistent circadian rhythm changes. Through the statistical analysis, it is found that SGNA recording has strong correlation with SCNA recording, and the correlation coefficient is 0.7, indicating that SCNA recording can replace SGNA recording to reflect the neural activities of stellate ganglion and the activity of cardiac sympathetic nerve (49).

Subsequently, Chan et al. (60) conducted 56 days of SGNA recording (direct measurement of stellate ganglion activity), SCNA recording, and HRV analysis on the canine model of MI. The absolute values of the correlation coefficients between integrated SGNA and SCNA were significantly larger than those between SGNA and HRV analysis based on time domain, frequency domain, and non-linear analyses, respectively, at baseline and after MI. The results showed that SCNA recording is better than HRV analysis in assessing cardiac sympathetic tone in dogs after MI. The feasibility of using SCNA recording to reflect cardiac sympathetic nerve activity is further verified (60). In addition, Doytchinova et al. (61) found that SCNA recording has a certain predictive value for the onset of ventricular tachycardia and ventricular fibrillation in dogs after MI and sudden cardiac death in rats with chronic renal failure (61).

Subcutaneous nerve activity recording avoids the huge trauma caused by SGNA recording that requires thoracotomy. By embedding the recording electrode in the subcutaneous tissue of the chest, SCNA recording can also complete the recording of nerve activity for more than 40 days for 24 h, reflecting the immediate SGNA and even cardiac sympathetic nerve activity (49, 61). However, it still has certain surgical trauma, which limits its clinical application. Jiang et al. (50) further explored the non-invasive recording of SKNA on the basis of SCNA recording.

#### Skin Sympathetic Nerve Activity

The histological evidence of human skin biopsy shows that there are abundant sympathetic nerves in the arteriovenous anastomosis, arrector pili muscle, and arterioles. Given the feasibility of SCNA record, Jiang et al. (50) further speculated that it is also feasible to directly record sympathetic nerve activities through the skin (Table 2).

Jiang et al. (50) directly attached the traditional ECG recording electrode to the dog’s chest skin for original signal recording, obtaining the signal of single lead ECG and SKNA by setting appropriate recording parameters and filtering parameters, and recorded SGNA as the gold standard (50, 51). The study found that in the resting state or stress state, SGNA and SKNA maintain a strong correlation, and the correlation coefficient is between 0.75 and 0.88, indicating that SKNA recording was consistent with SGNA recording (50). Doytchinova et al. (62) further verified the feasibility of SKNA recording on the recruited healthy volunteers and clinical patients. In total, nine healthy volunteers received cold-water stress test and Valsalva action successively. After the cold-water stress test began, the recorded SKNA signal increased significantly, and the subjects’ heart rate accelerated secondarily. After the Valsalva action, the SKNA signal of the subjects decreased rapidly, followed by a decrease in heart rate (62). In nine patients who underwent bilateral stellate ganglion block, the researchers found that SKNA signal was decreased by 63% from baseline after lidocaine injection into bilateral stellate ganglion (51). These studies further verified the feasibility of the SKNA recording to reflect the neural activity of stellate ganglion and even the activity of cardiac sympathetic nerve. Kumar et al. (63) further applied SKNA recording in patients with various CVDs, such as vasovagal syncope, heart failure with decreased ejection fraction, paroxysmal atrial fibrillation, and long QT interval syndrome to explore the role of cardiac sympathetic nerve activity in the pathogenesis and development of these diseases (64–69).

Skin sympathetic nerve activity can record cardiac sympathetic nerve non-invasively and continuously for 24 h. There are also several limitations to its usage, patients’ daily activities and even body movements have a great impact on the quality of recorded signals (50). Of note, Xing et al. (70) recently found a system-level modification by combining a commercial analog front end chip with a low-noise first-stage amplifier and an adaptive power-line-interference (PLI) filter and outliers clipping may reduce the system noise floor and reject the PLI and motion artifacts in the signal. The performance and effectiveness of this system have been verified in the laboratory experiment and clinical experiment (70). This may contribute to the popularization of SKNA. In addition, the parameters for SKNA are also limited to average SKNA (aSKNA, average sympathetic nerves activity during selected period, several min to h) and SKNA burst (number of sympathetic nerves burst discharges after excitation during the selected period). Due to the lack of large sample clinical studies, a well-established standard is warranted (50, 51).

## Conclusion

Sympathetic nerve dysfunction engages in the pathophysiological process of coronary heart disease, hypertension, heart failure, arrhythmia, and other CVDs. It is necessary to evaluate cardiac sympathetic activity. Generally speaking, these cardiac sympathetic activity assessment methods can be divided into three levels. Firstly, to evaluate the global sympathetic activities by plasma catecholamine levels, blood pressure variability, and so on. The evaluation results obtained by these methods may not be consistent with the true cardiac sympathetic activity state, which needs to be combined with other clinical evidence. Secondly, using HRV, cardiac neurotransmission imaging with SPECT and PET or SKNA evaluates the average state of sympathetic activity during a selected period of time. Thirdly, to monitor immediate sympathetic nerve activity by SKNA, which is impossible for other assessment methods.

What is noteworthy is that these cardiac sympathetic activity assessment methods are all based on the anatomy and physiology of the heart, especially the sympathetic innervation and the sympathetic regulation of the heart. Therefore, the depth of our understanding of the sympathetic innervation and sympathetic regulation of the heart determines the approaches we can take to evaluate cardiac sympathetic activity. Moreover, the development of cardiac neurotransmission imaging with SPECT, PET, and SKNA gives us a hint that technological advances and discipline overlapping are the important driving forces for improving cardiac sympathetic activity assessment methods.

## Author Contributions

JL wrote the manuscript. LZ modified and polished the manuscript. Both authors contributed to the article and approved the submitted version.

## Conflict of Interest

The authors declare that the research was conducted in the absence of any commercial or financial relationships that could be construed as a potential conflict of interest.

## Publisher’s Note

All claims expressed in this article are solely those of the authors and do not necessarily represent those of their affiliated organizations, or those of the publisher, the editors and the reviewers. Any product that may be evaluated in this article, or claim that may be made by its manufacturer, is not guaranteed or endorsed by the publisher.
